# Can trans‐generational experiments be used to enhance species resilience to ocean warming and acidification?

**DOI:** 10.1111/eva.12391

**Published:** 2016-07-06

**Authors:** Leela J. Chakravarti, Michael D. Jarrold, Emma M. Gibbin, Felix Christen, Gloria Massamba‐N'Siala, Pierre U. Blier, Piero Calosi

**Affiliations:** ^1^ Département de BiologieUniversité du Québec à RimouskiRimouskiQCCanada; ^2^ College of Marine and Environmental SciencesJames Cook UniversityTownsvilleQLDAustralia; ^3^ Laboratory for Biological GeochemistrySchool of Architecture, Civil and Environmental EngineeringÉcole Polytechnique Fédérale de LausanneLausanneSwitzerland; ^4^ Dipartimento di Scienze della VitaUniversità di Modena e Reggio EmiliaModenaItaly

**Keywords:** acclimation, climate change, conservation, global change, parental effects, phenotypic plasticity, restoration, selection

## Abstract

Human‐assisted, trans‐generational exposure to ocean warming and acidification has been proposed as a conservation and/or restoration tool to produce resilient offspring. To improve our understanding of the need for and the efficacy of this approach, we characterized life‐history and physiological responses in offspring of the marine polychaete *Ophryotrocha labronica* exposed to predicted ocean warming (OW: + 3°C), ocean acidification (OA: pH −0.5) and their combination (OWA: + 3°C, pH −0.5), following the exposure of their parents to either control conditions (*within‐generational exposure*) or the same conditions (*trans‐generational exposure*). Trans‐generational exposure to OW fully alleviated the negative effects of within‐generational exposure to OW on fecundity and egg volume and was accompanied by increased metabolic activity. While within‐generational exposure to OA reduced juvenile growth rates and egg volume, trans‐generational exposure alleviated the former but could not restore the latter. Surprisingly, exposure to OWA had no negative impacts within‐ or trans‐generationally. Our results highlight the potential for trans‐generational laboratory experiments in producing offspring that are resilient to OW and OA. However, trans‐generational exposure does not always appear to improve traits and therefore may not be a universally useful tool for all species in the face of global change.

## Introduction

Global environmental changes are occurring at an unprecedented rate compared with similar events in the geological past (IPCC, 2013). There is concern that natural rates of acclimation and/or adaptation will be insufficient for organisms to keep up. Indeed, growing evidence suggests that both ocean warming (OW) and ocean acidification (OA) will have negative impacts on the majority of marine metazoan species (Kroeker et al. 2013; Wittmann and Pörtner 2013), making them more susceptible to local and global extinction events (Cheung et al. 2009; Pandolfi et al. 2011; Bellard et al. 2012). Drastic measures proposed to mitigate the effects of OW and OA (hereafter collectively referred to as global change) include carbon capture and subterranean storage and solar radiation management. Such methods are controversial and are high risk (Vaughan and Lenton 2011; Blackford et al. 2014; Mathesius et al. 2015) and their implementation lags far behind the rate at which global change is occurring (Aswani et al. 2015).

The human‐assisted acceleration of naturally occurring evolutionary processes has been proposed as an alternative and direct form of intervention to develop stress‐resilient populations to withstand global change (Aswani et al. 2015; Van Oppen et al. 2015). This concept includes the parental exposure of populations to relevant global change conditions as a method to induce trans‐generational plasticity (TGP) and/or to rapidly select for offspring that are more resilient to future ocean conditions (Van Oppen et al. 2015). TGP occurs through nongenetic parental effects, whereby environmental conditions experienced by the parental generation influences the phenotype of subsequent generations (Mousseau and Fox 1998). Positive TGP can occur through maternal effects, such as increases in egg provisioning by the mother, or through epigenetic changes that involve heritable alterations in gene expression and function (Bonduriansky et al. 2012). In contrast to TGP, natural selection can act upon genetic variation so that genotypes better able to survive and reproduce under a new set of environmental conditions increase in proportion, which can lead to evolutionary adaptation (Darwin 1859; Hoffmann and Sgrò 2011). Adaptation is usually a slower process than TGP. However, the induction of rapid environmental change can cause fast rates of adaptation through differential survival or reproductive success (Hermisson and Pennings 2005); theoretically, this can occur in a single generation (Christie et al. 2012).

The use of trans‐generational exposure is already applied in aquaculture to enhance the performance of offspring (e.g. Panagiotaki and Geffen 1992; Gile and Ferguson 1995; Saillant et al. 2001). Furthermore, harnessing trans‐generational effects in captive‐reared populations could be successful as a conservation or restoration tool for endangered fish species (Evans et al. 2014). More recently, laboratory experiments have shown that trans‐generational effects can mediate the negative impacts of future global change on offspring. For example, TGP was responsible for three fish species performing better under OW conditions, through improvements in aerobic scope or growth, if their parents had also experienced OW (Donelson et al. 2012; Salinas and Munch 2012; Shama et al. 2014, 2016). Similarly, parents exposed to OA produced offspring with faster growth and development under OA than those reared under control conditions in a fish and oyster species, respectively (Miller et al. 2012; Parker et al. 2012). On the other hand, trans‐generational selection could have accounted for the mediation of negative OA impacts on fecundity in copepods (De Witt et al. 2015; Thor and Dupont 2015).

Although the outcome of laboratory, trans‐generational exposure on offspring performance is increasingly being investigated in the context of global change (e.g. Shama et al. 2014, 2016; Parker et al. 2015; Thor and Dupont 2015), most studies have focused on the performance of either the early life stages of offspring (Munday 2014) or on only the adult stages of offspring (e.g. Massamba‐N'Siala et al. 2014; Thor and Dupont 2015). It therefore remains unclear whether responses derived from trans‐generational exposure, often observed in the early life stages, are also observed in adults (but see Parker et al. 2015). Furthermore, few studies have examined the effectiveness of trans‐generational exposure to simultaneous OW and OA conditions (but see Vehmaa et al. 2012; Putnam and Gates 2015). Understanding the effects of trans‐generational exposure across juvenile and adult life stages, under multiple global change scenarios, is vital to assess the role that human‐assisted, trans‐generational effects can play in conservation and/or restoration.

In order to thoroughly test whether laboratory, trans‐generational exposure can improve species' resilience to future global changes, we conducted an experiment using an emerging marine model for multigenerational studies, the polychaete *Ophryotrocha labronica* La Greca and Bacci (1962). This species has a short generation time (ca. 17 day at 27°C; Åkesson 1976) and can be easily reared for multiple generations under controlled laboratory conditions, with the possibility of gaining information on a number of life‐history (Massamba‐N'Siala et al. 2011, 2012; Rodriguez‐Romero et al. 2015) and physiological (Massamba‐N'Siala et al. 2012, 2014) traits. Specifically, individuals of *O. labronica* were reared from hatching to spawning under control (C – 27°C, pH 8.05), OW (30°C, pH 8.05), OA (27°C, pH 7.60) or OW and OA combined (OWA; 30°C, pH 7.60) conditions, thus becoming the parental generation. Their offspring were transplanted into the same treatment conditions (C–C, OW–OW, OA–OA and OWA–OWA) to make up the trans‐generational exposure group, while offspring from control‐reared parents were also transplanted into each treatment condition to make up the within‐generational exposure group (C–OW, C–OA or C–OWA). Key, fitness‐related life‐history traits, including hatching event success, juvenile growth rate and survival to sexual maturity, fecundity, egg volume and female maximum size (Rodriguez‐Romero et al. 2015), were compared in offspring exposed within‐ and trans‐generationally. Physiological proxies for metabolic activity and function, and citrate synthase and electron transport system activities and reactive oxygen species (ROS) production (Moyes et al. 1997; Rivera‐Ingraham et al. 2013; Smith et al. 2014; Schmidlin et al. 2015) were also measured in offspring to provide a mechanistic underpinning to any observed differences in life‐history responses (Sibly and Calow 1986).

## Materials and methods

### Organism collection

*Ophryotrocha labronica* is a small (≈4 mm maximum length), widespread, benthic polychaete commonly found in coastal, organically enriched environments such as harbours and lagoons (Prevedelli and Simonini 2003; Simonini et al. 2009). *Ophryotrocha labronica* is gonochoric and offspring directly develop within a week from tubular egg masses that are produced semi‐continuously. Individuals used in this study originated from a population of *circa* 100 polychaetes collected in January 2014 from Porto Empedocle harbour, Italy (37°17′4″ N, 13°31′3″ E). The individuals were kept in small glass dishes (ø = 7 cm, depth = 3 cm) in artificial seawater (Aquarium Sea Salt Mixture, Instant Ocean^®^, Blacksburg, VA, USA) at a constant temperature (mean ± SD, 27 ± 0.5°C), salinity (35 ± 2) and pH (8.05 ± 0.1), under a 12: 12 light: dark photoperiod. Worms were fed weekly *ad libitum* with minced spinach (Massamba‐N'Siala et al. 2012), always at a frequency and quantity that permitted all of the spinach to be eaten. Weekly water changes prevented undesired fermentation and accumulation of excreta and maintained stable oxygen levels (always >70%).

### Experimental conditions and set‐up

In order to investigate both within‐ and trans‐generational responses to OW, OA and their combination (OWA), we exposed *O. labronica* to four experimental treatments: control (C): pH 8.05, 27°C, OW: pH 8.05, 30°C, OA: pH 7.60, 27°C and OWA: pH 7.60, 30°C. The pH chosen for the OA and OWA treatments represented values currently experienced intermittently by shallow water coastal organisms, but which are expected to occur chronically in the future (Thomsen et al. 2010; Shaw et al. 2012; Duarte et al. 2013; IPCC 2013; Melzner et al. 2013). The temperature chosen for OW conditions represented a + 3°C increase from our experimental control conditions (IPCC, 2013).

The experimental treatments were maintained using a carbon dioxide (CO_2_) and temperature manipulation set‐up (Figure S1), similar to that described in Rodriguez‐Romero et al. (2015). Briefly, the system comprised of four deep trays (68 × 48 × 26 cm) that were half‐filled with deionized water. Two trays were heated to 27°C and the remaining two heated to 30°C with aquarium heaters (100 W Hydor, Sacramento, CA, USA). Power head pumps (Koralia Circulation and Wave Pump; Hydor) were placed behind each heater to ensure good water circulation in the trays and a homogeneous temperature. In addition, each tray was covered with a Perspex sheet to minimize the evaporation of deionized water and thus limit thermal fluctuations.

Each tray contained four airtight tubs (5 L, Figure S1); two perfused with CO_2_‐enriched air and two with control air. CO_2_‐enriched air was achieved by mixing ambient air with pure CO_2_ using tubing and gang valves. The actual partial pressure of CO_2_ (*p*CO_2_) in the gas mixture was measured using a CO_2_ analyser (LI‐840A; Li‐Cor, Lincoln, NE, USA) to manually ensure the levels of injected CO_2_ were stable. Control air was achieved by pumping ambient air through a 1 m sodium hydroxide (NaOH) scrubbing solution, to maintain the control pH condition. Control and CO_2_‐enriched air were supplied to each tub *via* an aquarium air pump (Mistral 4000; Aqua Medic, Bissendorf, Germany) through tubing connected to decapped microcentrifuge tubes inserted through the top of each tub.

Each tub held two, six‐well culture plates (Corning Ltd, Sunderland, UK, Figure S1) and each plate had the capacity to house three broods of offspring and three adult pairs (*n *=* *3 replicate pairs/broods *per* plate, 12 replicate pairs/broods total *per* treatment, Figure S1). Plates were covered with a breathable film (Aeraseal; Alpha Laboratories Ltd, Eastleigh, UK) to enable gas exchange, while minimizing evaporation inside the culturing wells, thus reducing temperature and salinity fluctuations.

### Seawater parameters

Temperature, pH and salinity were measured daily in one random well of each of the six‐well culture plates (*n *= 1372; Table S1), coinciding with daily water changes. Weekly, 50 mL of seawater samples from each plate were taken to quantify dissolved inorganic carbon (DIC) content (*n *= 157; Table S1) using a Dionex Ion Chromatography System, equipped with an AS40 automated sampling machine, a DS6 heated conductivity cell (Thermo Fisher Scientific, Waltham, MA, USA), and using the chromeleon client 6.80 software (Actuate Corporation, San Mateo, CA, USA). The remaining carbonate system parameters were subsequently calculated with the program CO_2_SYS (Table S1) using measured salinity, pH and DIC values (Pierrot et al. 2006), using Mehrbach constants (Mehrbach et al. 1973) as refitted by Dickson and Millero (1987).

### Experimental design

The effects of within‐ and trans‐generational exposure to OW, OA and OWA were determined using an experimental design similar to that used by Miller et al. (2012) and Allan et al. (2014). A summary diagram is shown in Figure S2.

Twelve breeding pairs (F0) were isolated from the laboratory culture and the second egg mass spawned by each pair was left to hatch. Previous experiments have indicated that the first egg mass is smaller than the subsequent egg masses, a relationship driven by the positive correlation between fecundity and body size (Berglund 1991; Massamba‐N'Siala et al. 2011). Therefore, to ensure a sufficient number of individuals for the experiment, F2 offspring were obtained from the second egg masses spawned by F1 pairs, while the first egg masses were removed after the determination of their size (see next section). Three days after hatching, *circa* 20 offspring (F1) from each replicate brood were moved into a plate contained within each of the four experimental treatments (Figure S2). Upon reaching sexual maturity, F1 individuals from each of the 12 broods were paired by crossing males and females from different wells (different broods) within the same culture plate to avoid inbreeding effects. The pairs were placed into new wells of the same plate (Figure S2). Adults that were not used to form pairs were kept as ‘spares’ for one generation before being discarded.

To determine the trans‐generational effects of exposure to OW, OA and OWA conditions on the life‐history and physiological performances of F2 individuals, ~20 hatchlings from each replicate brood were transplanted into the same conditions as their parents. This trans‐generational treatment was characterized as OW–OW, OA–OA and OWA–OWA (Figure S2). To determine the within‐generational effects of exposure to OW, OA and OWA conditions on F2 individuals, ~20 hatchlings from each control‐conditioned parents were transplanted on the day of their hatching into each of the four experimental treatments. These within‐generationally exposed individuals were characterized as C–C, C–OW, C–OA and C–OWA treatments (Figure S2).

### Determination of life‐history traits

Hatching event success, juvenile growth rate and survival to sexual maturity, fecundity, egg volume and female maximum size represent key life‐history traits for *O. labronica* and were chosen as proxies for performance under the different treatment conditions.

The eggs spawned by some F1 pairs under the OW and OWA treatments did not hatch. In this situation, new F1 pairs were formed by randomly choosing spare individuals to obtain viable offspring for the F2 generation. All second attempts were successful. Therefore, hatching event success was recorded as the percentage of initial number of F1 pairs, out of the 12 pairs formed that were able to produce at least 20 F2 offspring and became an important proxy for selection. The act of randomly forming of new pairs did not bias our results as individuals not able to reproduce in nature would also not give any contribution to the next generation.

Juvenile growth rates of F2 offspring were used as a proxy for juvenile development and were determined 7 days after hatching by counting the number of chaetigers (chaetae‐bearing segments) of six randomly selected individuals from each of the 12 broods in each treatment (*n *= 72), using a light microscope (MS5, Leica, St. Gallen, Switzerland) at ×2.5 magnification. Juvenile growth rates were standardized to the number of chaetigers added *per* day (chaetigers day^−1^).

Survival to sexual maturity was determined to understand whether any differential selection occurred between treatments and transplants, from hatching to adulthood. Survival was calculated by counting the number of F2 individuals from each brood (*n *= 12) remaining in each well once they had reached sexual maturity and was expressed as the percentage of the total number of individuals moved on the day of hatching. Sexual maturity was determined as the earliest point at which developing eggs could be observed within the coelomic cavity of the females. Reaching sexual maturity coincided with the formation of the F2 pairs.

F2 pairs were kept in their assigned treatment until the production of their third egg mass. If a male partner died during the experiment, it was replaced with a spare male from the same treatment to ensure that females' spawning was unaffected (Massamba‐N'Siala et al. 2011, 2012). Fecundity was measured by counting the total number of eggs produced in egg masses one and three for each replicate pair in each treatment (*n *= 12), using a digital microscope camera (14 MP; Omax, Bucheon, South Korea). Data are not available for the second egg masses because these were required to maintain our culture for further multigenerational studies and manipulations would have led to significant levels of mortality.

*Ophryotrocha labronica* colonizes temporally and spatially fluctuating, unpredictable environments and is described as an r‐strategist, that is characterized by short generation times, high fecundity and early onset maturity (MacArthur and Wilson 1967). For these organisms, early reproduction is crucial, so we used images obtained for egg mass one to determine egg volume, which is broadly described as a proxy for egg quality (but see Moran and McAlister 2009; Allen and Marshall 2014). Specifically, we measured the longest and shortest *radii* of 10 eggs from each egg mass, using the software imagej (https://imagej.nih.gov/ij/), and applied them to the following formula:

$$V=\frac{4}{3}\pi \;A^{2}B$$

where *A* is the short radius, and *B* is the long radius (Simonini and Prevedelli 2003). Egg volume was expressed as × 10^−3^ mm^3^.

Adult female size (number of chaetigers) is directly correlated to fecundity in *O. labronica*, so the size of each female was recorded at the first, second and third reproductive events and was subsequently used to identify the maximum female size (*n *= 12).

### Determination of physiological traits

Physiological traits were measured in adult females to determine the mechanisms behind any changes in adult life‐history parameters. These measurements included citrate synthase (CS) activity as a proxy for mitochondrial density (Moyes et al. 1997) and a relative measurement of aerobic metabolism, as well as the activity of the Electron Transport System (ETS), an enzyme complex located on the inner mitochondrial membrane widely accepted as a marker for maximum mitochondrial capacity (Schmidlin et al. 2015). Additionally, a by‐product of aerobic metabolism, ROS were measured in adult females (Rivera‐Ingraham et al. 2013; Smith et al. 2014). The production of ROS is normal during metabolic processes and has important cellular functions in low quantities; however, when produced in excess, ROS can cause significant cellular damage and disrupt physiological function (Lesser 2006).

Once the third egg mass produced by F2 pairs had been spawned, four spare females from the same brood and treatment (*n *= 12) were transferred into a 1.5‐mL microcentrifuge tube and frozen at −80°C for later CS and ETS enzyme analyses. In addition, a randomly selected female was isolated from each brood, of each treatment, and prepared for confocal microscopy in order to measure ROS production and mitochondrial density (see below, *n* = up to 12).

We calculated the ratio of CS to ETS activities. CS is a soluble enzyme in the matrix whose activity should correlate with mitochondrial volume, ETS expresses the activity of enzymatic complexes embedded in the inner membrane (proportional to surface area) and any divergence between CS and ETS activity thus expresses differences in mitochondrial morphology or structural and functional organization. Finally, we estimated the production of ROS and these values were normalized to mitochondrial density.

### Determination of citrate synthase and electron transport system activities

Citrate synthase and ETS activities were normalized to protein content. Measurements were taken using a UV/VIS microplate spectrophotometer (Perkin Elmer Envision, Foster City, CA, USA). Samples were homogenized in 120 μL of ice‐cold 100 mm phosphate buffer, 20 mm EDTA (pH 8.00). CS, ETS and protein content were quantified using the same homogenate. CS activity was measured in 0.1 mm 5,5′‐dithiobis (2‐nitrobenzoic acid) (DTNB), 0.1 mm acetyl‐CoA and 0.15 mm oxaloacetate (pH 8.00). CS activity was measured in triplicate at 27°C and was calculated from the increase in absorbance at 412 nm over 3 min (*ε*_412_ = 13.6 mL cm^−1^ μmol^−1^), caused by the reduction in DTNB (Thibeault et al. 1997). ETS activity was measured in 0.85 mm *β*‐Nicotinamide adenine dinucleotide, reduced disodium salt hydrate, 2 mm Iodonitrotetrazolium chloride (INT) and 0.03% Triton^™^ X‐100 (Sigma‐Aldrich, Mississauga, ON, Canada) (pH 8). Activities were measured in triplicate at 27°C by following the increase in absorbance due to the reduction in INT at 490 nm for 4 min (*ε*_490_ = 15.9 mL cm^−1^ μmol^−1^) (Bergmeyer et al. 1983). Total protein content was determined on homogenates using the bicinchoninic acid method (Smith et al. 1985).

### Quantification of reactive oxygen species levels

Fluorescent confocal microscopy is a powerful tool for estimating ROS production (Rivera‐Ingraham et al. 2013; Smith et al. 2014) and mitochondrial density (Dingley et al. 2010). Here, we use two fluorescent probes, CellROX Deep Red and MitoTracker Green FM (Life Technologies, Invitrogen, Carlsbad, CA, USA). The former dye fluoresces when it is oxidized by ROS, while the latter becomes fluorescent after binding to a specific subset of proteins found in mitochondria (Presley et al. 2003). In order to improve the quality of the images, specimens were starved for 2d before imaging. Individuals were incubated in treatment water with 5 μm CellROX Deep Red and 400 nm of MitoTracker Green, contained within an opaque box to avoid dye degradation, and placed on a rotating plate set to 60 rpm. After 30 min, the samples were thoroughly washed in clean treatment water to remove any residual dye, and placed in a glass‐bottom Petri dish for imaging. Excess water was then removed, and each individual was immobilized with a drop of slowing solution (Detain Wards Protist slowing agent; Wards, Rochester, NY, USA). 3D Z‐stack images were taken at ×20 magnification under a confocal laser‐scanning microscope (LSM 700, Carl Zeiss, Oberkochen, Germany) using the zen 2009 software. A 1.5 μm stack width was deemed optimal as this produced a minimum of 15 slices. Chaetigers 4, 5, 8 and 9 were imaged for each individual. Analysis was conducted with imagej (National Institute for Health, Bethesda, MD, USA) using the ‘Z‐project’ command. The first 10 slices were used for analysis. Individual segments were enclosed manually, and fluorescence integrated density (selected area × mean intensity) was quantified for both dyes simultaneously. ROS production was normalized to mitochondrial density by dividing fluorescence values for ROS production by fluorescence values for mitochondrial density. All chemicals used for these assays, unless otherwise mentioned, were purchased from Sigma (Mississauga, ON, Canada) or VWR (Mississauga, ON, CA).

### Statistical analyses

The effects of parental experimental conditions on the percentage of successful hatching events to produce F2 offspring were analysed with chi‐squared tests (*χ* ^2^), where 100% of the initial 12 pairings were expected to be successful.

To analyse the effects of within‐ and trans‐generational exposure to each experimental treatment (OA, OW and OWA), we compared the effects of control conditions, within‐generational exposure and trans‐generational exposure (e.g. C‐C *vs*. C‐OW *vs*. OW‐OW) on *O. labronica* life‐history and physiological responses. We used GLM tests with ‘treatment’ as a fixed factor, ‘tray’ as a random factor nested within ‘treatment’ and ‘tub’ as a random factor nested within ‘treatment × tray’. Maximum female size was used as covariate for fecundity data.

Random factors that had no significant effect (*P *> 0.05) were systematically removed one at a time (highest *P*‐value first) from the analysis, until only significant factors and the main effects were left. The random factor ‘tub’ was found to have significant effects on some traits (min *F*_8,32_ = 2.16, *P *= 0.042). However, as we found no significant differences in measured water parameters between tubs within a treatment (max *F*_8,1357_ = 1.49, *P* = 0.157), we considered the observed ‘tub’ effects as mainly driven by differences in the genetic material, and consequently ‘tub’ was removed from the analyses.

Assumptions for normality of distribution of the data and homogeneity of the variance were verified using the Kolmogorov–Smirnov and the Levene tests, respectively. When data did not meet the assumption of normality or homogeneity, the residuals from each analysis were tested against the study factors using GLM tests. In all cases, no significant relationships were detected (*P *> 0.05), supporting the use of GLMs. All pairwise comparisons were conducted using *post hoc* Tukey's tests. Analyses were conducted using Minitab 16.

## Results

### Hatching event success

**Figure 1:**
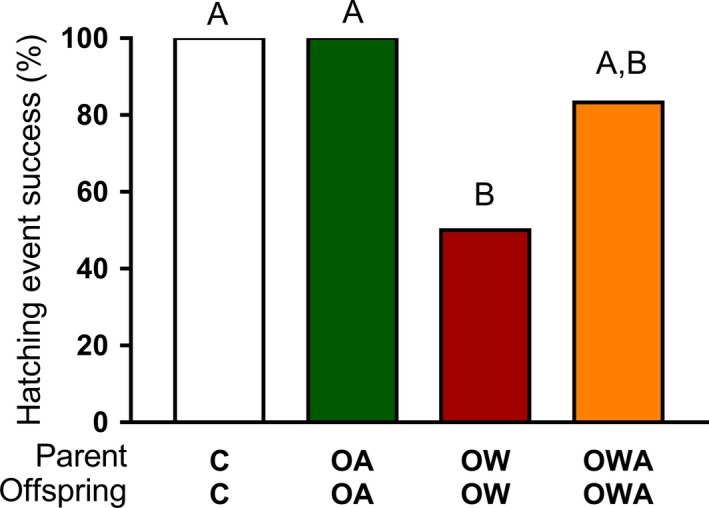
The effect of exposure to elevated temperature and low pH/high *p*CO_2_ in isolation and combined on the percentage of successful hatching events in the polychaete worm *Ophryotrocha labronica*: control (C: 27°C, pH 8.05), ocean warming (OW: 30°C, pH 8.05), ocean acidification (OA: 27 °C, pH 7.60) and their combination (OWA: 30°C, pH 7.60). The bar plot represents the percentage of successful hatching events (i.e. spawning events that produced viable F2 offspring) from the original pairs used to generate F1 (*n *= 12). Letters above the bar plots represent statistically significant differences (*P < *0.05) between treatments.

All hatching events of egg masses produced by F1 pairs were successful in the control and OA treatments (Fig. 1), while a significantly lower percentage of egg masses (50%), hatched successfully following exposure to OW (Fig. 1, *χ* ^2^ = 6.000, d*f* = 1, *P *<* *0.005). Under OWA conditions, 83% (10 of 12) of hatching events were successful, which was statistically comparable to those obtained for all of the other treatments (Fig. 1, max *χ* ^2^ = 3.333, d*f* = 1, *P* = > 0.005).

### Juvenile growth

**Figure 2:**
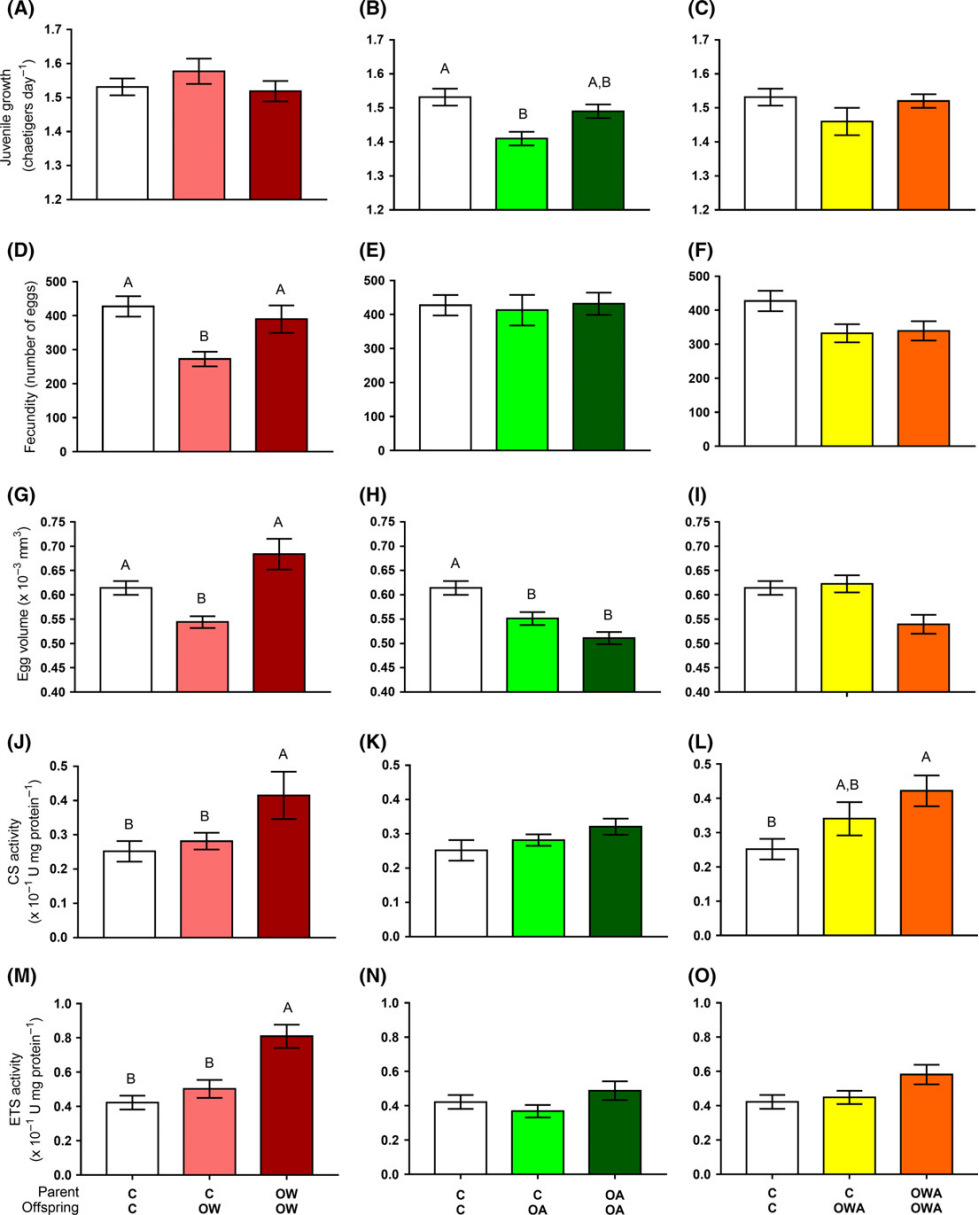
The effect of within‐ and trans‐generational exposure to elevated temperature and low pH/high *p*CO_2_ in isolation and combined [control (C), ocean warming (OW), ocean acidification (OA) and their combination (OWA)] on (A–C) mean juvenile growth rate, (D–F) total fecundity, (G–I) egg volume, (J–L) citrate synthase (CS) activity and (M–O) electron transport system (ETS) activity, in *Ophryotrocha labronica* after the parental generation had been reared under either the same (trans‐generational exposure: C–C, C‐OW, C‐OA, C‐OWA) or control conditions (within‐generational exposure: C‐OW, C‐OA, C‐OWA). Different letters above the bar plots represent statistically significant differences (*P < *0.05). Error bars represent mean ± SE.

Mean juvenile growth rates were significantly slower under exposure to OA (Table S3). Specifically, juveniles grew slower than the control individuals when exposed to OA for one generation (i.e. within‐generational exposure; Fig. 2B). However, following trans‐generational exposure to OA, the mean juvenile growth rate was comparable to that of the control and within‐generationally exposed individuals (Fig. 2B). Mean juvenile growth rates did not significantly change following either within‐ or trans‐generational exposure to OW or OWA (Table S4, S5, Fig. 2A,C).

### Juvenile survival to sexual maturity

The mean percentage of individuals surviving to sexual maturity was significantly lower under exposure to OW (Table S4), but this only occurred when comparing survival in the within‐generationally exposed group to those trans‐generationally exposed. Survival was significantly lower in the former compared with the latter, although neither significantly differed from the control (Table S4). The percentage of individuals surviving to sexual maturity was not significantly impacted by either within‐ or trans‐generational exposure to OA and OWA when compared to each other, or to individuals in control conditions (Table S3, S5).

### Fecundity

Individuals exposed within a generation to OW produced significantly fewer eggs than control individuals (Table S4, Fig. 2D). In contrast, trans‐generational exposure to OW led to a significant increase in mean fecundity to levels comparable to the control, and greater than those measured in individuals exposed within a generation to OW (Fig. 2D). Fecundity was not significantly affected by exposure to OA or OWA (Table S3, S5, Fig. 2E,F).

### Egg volume

Mean egg volume was significantly lower in individuals exposed within a generation to both OW and OA (Tables S4, S3), relative to the control (Fig. 2G,H). Trans‐generational exposure to OW fully alleviated the decline in egg volumes, so that levels were comparable with the control (Fig. 2G). On the other hand, in individuals exposed over successive generations to OA conditions, the mean egg volume was still smaller than that of individuals exposed to control conditions, and comparable to that reported for individuals exposed within a generation to OA (Fig. 2H). Differently, mean egg volumes produced by individuals exposed to OWA within and across generations were comparable to controls (Table S5, Fig. 2I).

### Citrate synthase and electron transport system activities

Mean citrate synthase (CS) activity was significantly higher in individuals exposed to OW (Table S4) but only following trans‐generational exposure (Table S4), with levels significantly greater than those measured in individuals exposed to both control or within‐generational OW conditions (Fig. 2J). Exposure to OWA resulted in a similar pattern, with a significantly higher mean CS activity measured in individuals exposed trans‐generationally to OWA when compared to those from the control (Table S5). Differently, mean CS activity of individuals exposed trans‐generationally to OA was statistically comparable to that measured in individuals exposed within a generation to OA (Fig. 2L).

Mean ETS activity showed the same response as mean CS activity under OW (Table S3, Fig. 2M), but was not affected by exposure to OA or OWA (Table S3, S5), with statistically comparable levels measured in individuals exposed to control, within‐ and trans‐generational conditions (Fig. 2N,O). The ratio of CS:ETS was not significantly affected by within‐ or trans‐generational exposure to OA, OW or OWA (Tables S3–S5).

### ROS production

Mean ROS production was also not significantly affected by exposure to OA, OW or OWA either over a single or successive generations (Tables S3–S5).

## Discussion

Our study shows that trans‐generational exposure to OW and OA is able to enhance performance across a wide range of key fitness‐related life‐history and physiological traits at both juvenile and adult life stages. Among the eight traits measured in *O. labronica*, three were negatively impacted by within‐generational exposure to either OW or OA. Following trans‐generational exposure, three traits were restored to control levels, two positively increased to levels greater than the control and only one trait remained negatively affected (Table 1). Surprisingly, the combined exposure to OW and OA (OWA) had no negative effect within or across generations. Overall, our results on single‐stressor exposure support the use of human‐assisted trans‐generational effects to enhance offspring resilience. However, the lack of a negative response when exposed to combined stressors suggests that trans‐generational exposure as a conservation or restoration tool may not be necessary in some cases; the benefits acquired from human‐assisted approaches are likely to be highly species or taxa specific.

**Table 1:**
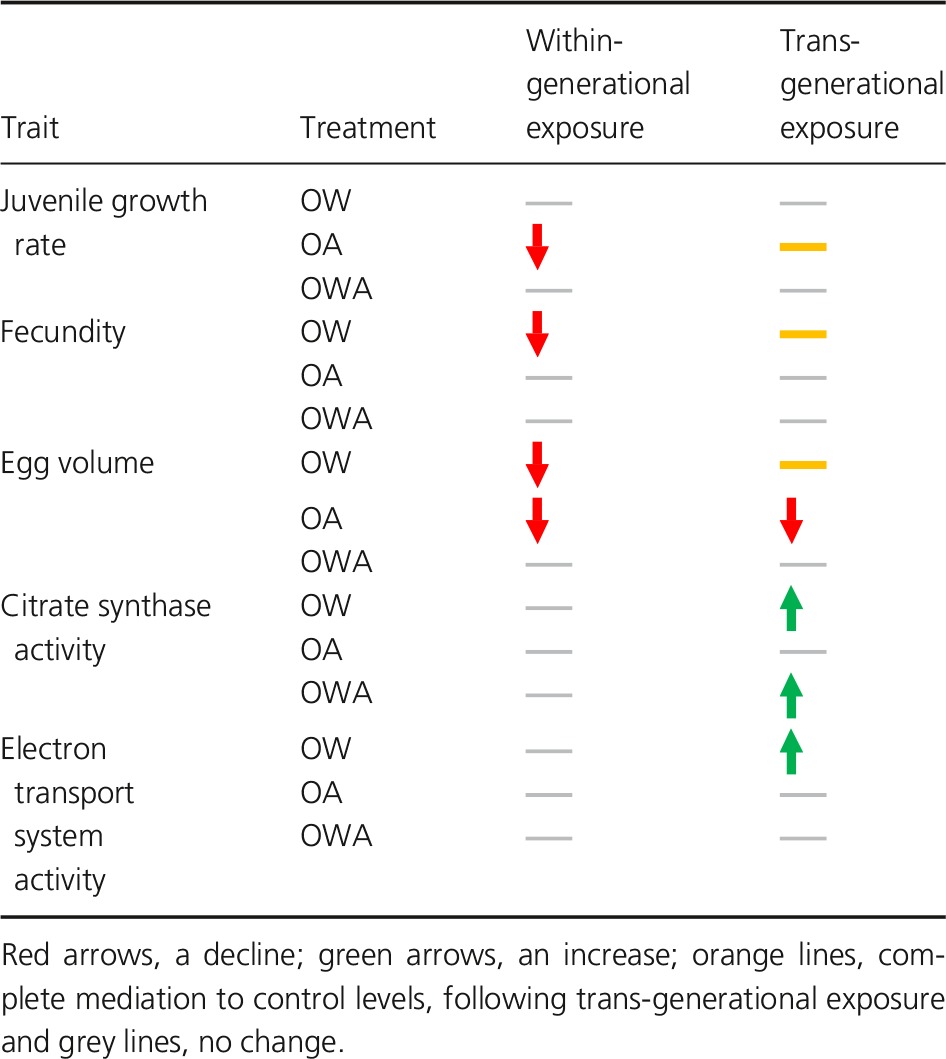
Direction of response for significantly affected traits in *Ophryotrocha labronica* following within‐ and trans‐generational exposure to ocean warming (OW), ocean acidification (OA) and their combination (OWA), relative to the control

### Trans‐generational exposure to ocean warming

Fecundity and egg volume decreased by 36% and 11%, respectively, after within‐generational OW exposure in *O. labronica*. However, following trans‐generational exposure, both traits were fully restored to levels comparable to the control (Table 1). Similar trans‐generational improvements under OW conditions have been observed in marine sticklebacks (Shama et al. 2014, 2016) and juvenile damselfish (Donelson et al. 2012), both accompanied by increases in metabolic performance and the former by changes in the expression of genes involved in mitochondrial respiration (Shama et al. 2016). Comparably, more than a 30% increase in metabolic activity, detectable in both mitochondrial CS and ETS activities, accompanied the full trans‐generational restoration of reproductive traits in our study (Table 1). A trans‐generational increase in metabolic activity is likely needed to support the increase in energetic demands required of living at a higher temperature, thus enabling reproductive traits to be maintained. The similar observations in CS:ETS ratios between treatments indicate that metabolic adjustments in *O. labronica* were likely to have arisen from quantitative modifications in mitochondrial biosynthesis, rather than alterations in mitochondrial structure or function.

Costs of increased metabolic activity on life‐history traits were not observed in this study; however, increased metabolic activity may not be sustainable in the longer term. Increases in mitochondrial activity can result in a greater production of ROS, which can cause oxidative damage to lipids, proteins and nucleic acids, ultimately impairing physiological functions (Stadtman 1992; Lesser 2006). Nevertheless, we did not observe a change in the net production of ROS under trans‐generational OW suggesting that antioxidant defences and/or repair systems were able to cope with metabolic adjustments in *O. labronica*. If, however, the Redox homeostasis is able to be maintained through upregulation of antioxidant defences, this could come at a cost to energetically expensive processes such as future reproduction, longevity and growth (Alonso‐Alvarez et al. 2004) perhaps occurring in successive generations.

Only 50% of egg masses hatched successfully when spawned under OW, implying lowered fertilization success and/or embryo survival in this study. Comparably, both fertilization and embryonic development were lowered above 26°C in the Sydney rock oyster *Saccostrea glomerata*, with no embryonic development at 30°C (Parker et al. 2009). Our observed improvements in reproductive traits for *O. labronica* exposed trans‐generationally to OW could have been as a result of selection at the gamete or embryonic stage such that only genotypes with greater metabolic capacities survived to adulthood. Indeed, previous studies have indicated that rapid selection under a variety of stressful environmental conditions can occur across only two generations through sufficiently high mortalities (Vidal and Horne 2009; Christie et al. 2012; Thor and Dupont 2015) or differential reproductive success (Donelson et al. 2012), such that genetic adaptation cannot be ruled out as a mechanism for improvements in offspring performance.

### Trans‐generational exposure to ocean acidification

Juvenile growth rates were reduced by 9.4%, when exposed to within‐generational OA, relative to the control in this study (Table 1). Reductions in growth and development following exposure to future OA conditions are common in the early life stages of marine metazoans (Kroeker et al. 2013), likely caused by increases in the energetic demand associated with maintaining homeostasis resulting from disruptions in intracellular acid–base balance and ion transport systems (Pörtner 2008; Melzner et al. 2009; Stumpp et al. 2012). We observed a partial restoration in juvenile growth rates (+5.1%) in individuals trans‐generationally exposed to OA, compared with those exposed within‐generationally (Table 1). Similar results were reported in the clownfish, *Amphiprion melanopus* (Miller et al. 2012) and in the oyster, *S. glomerata* (Parker et al. 2012), where trans‐generational OA exposure increased the size of offspring by 6% and 10%, respectively. Juvenile growth rates are key measures of fitness across r‐strategy species such as *O. labronica*, relying on fast development for early reproduction to maximize fecundity (Charlesworth 1980). Thus, the ability to mitigate the negative effects of OA on juvenile growth and development is critical in maintaining population fitness.

Trans‐generational plasticity most likely explains our observations of a partial increase in mean juvenile growth rate under trans‐generational OA exposure. This is assumed because hatching event success was 100% in OA conditions and subsequent survival to sexual maturity was comparable to the control; little to no natural selection is expected to have occurred. TGP can involve the maternal transmission of nutritional material to offspring (Bonduriansky et al. 2012), as shown in oysters, where an increase in developmental rates correlated with increases in egg size (Parker et al. 2012). However, in our study, the mean egg volume produced by mothers under OA was *circa* 10% lower than the control, suggesting that the partial improvement in their offspring's growth rate was unlikely to be as a result of better maternal provisioning. Trans‐generational improvements may also occur through epigenetic inheritance, whereby phenotypic variations are transmitted across generations without any variations in DNA (Jablonka and Raz 2009). Epigenetic effects have been attributed to improving offspring performance in the clownfish *A. melanopus* trans‐generationally exposed to OA, likely through the activation of more efficient physiological pathways (Miller et al. 2012). Despite this, mean CS and ETS activities were not altered by trans‐generational exposure to OA in *O. labronica*; thus, we cannot explain increased juvenile growth rates through epigenetic changes involved in metabolism.

Egg volume did not improve following trans‐generational OA exposure, with eggs remaining smaller (−17%) than the control. Despite this, fecundity was maintained at levels comparable to the control (Table 1). In contrast, the copepod *P. acuspes* experienced no reductions in egg size, but a 29% reduction in fecundity after trans‐generational OA exposure (Thor and Dupont 2015). This suggests that different life‐history strategies under stressful conditions can exist, such that a trade‐off in reproductive resource allocation may occur between maximizing maternal fitness, through maintaining reproductive output, with the ability to offer sufficient provisioning for their offspring, through maintaining egg quality (and size) (Uller 2008). Nevertheless, the decrease in *O. labronica* egg volume did not impact the performance of offspring, and we observed an increase in juvenile growth rates. For this reason, we cannot assume that the failure to restore egg volumes under trans‐generational exposure to OA would have resulted in inferior progeny in the next generation. Indeed, a decline in yolk sac area, a proxy for maternal provisioning, came at no cost to hatchlings of the clownfish *A. melanopus* exposed to OA (Miller et al. 2013).

### Exposure to combined ocean warming and acidification

Neither within‐ nor trans‐generational exposure to combined OW and OA negatively affected any measured traits in *O. labronica*. Where negative, within‐generational effects of OW were observed in isolation, the addition of OA acted to neutralize them, and *vice versa*. The only exception was represented by a 40% increase in mitochondrial CS activity following trans‐generational exposure to OWA compared with those reared under control conditions (Table 1), likely to be a requirement to support the increase in energy demands of living at an elevated temperature, as observed under OW conditions in isolation.

Most studies have observed negative effects of multiple global change drivers in marine metazoans exposed within a generation (Kroeker et al. 2013), in contrast with our results. A limited number of studies have investigated the effects of trans‐generational exposure to simultaneous OW and OA on offspring performance; the tropical coral, *Pocillopora damicornis* produced smaller larvae but with increased metabolic rates (Putnam and Gates 2015), while the copepod *Acartia* sp. had a reduced hatching success (Vehmaa et al. 2012). However, it is important to note that in both experiments parental exposure to OWA started at the adult, reproductively active stage, while in our study we exposed the parental generation from hatching through to adulthood. The duration and stage of parental exposure may alter the trans‐generational performance of offspring, *via* cumulative carry‐over effects across life stages (see Dupont et al. 2013; Suckling et al. 2014).

Hatching event success in *O. labronica* was 100% under OA, 50% under OW and 83% under OWA conditions (Table 1). This suggests that OA can partially offset the negative impact of OW on either fertilization success and/or embryo survival, when combined in this species. Negative effects of OW on hatching success are perhaps not surprising; fertilization success declined above a temperature threshold in the rock oyster *S. glomerata* (Parker et al. 2009), while embryonic development was reduced by exposure to OW in the tropical coral *Diploria strigosa* (Bassim et al. 2002) and the sea urchin *Heliocidaris erythrogramma* (Byrne et al. 2009). In terms of OA, no negative effects of low pH on fertilization or embryonic development were observed in sea urchins, oysters and mussels (Kurihara 2008; Byrne et al. 2009; Havenhand and Schlegel 2009). This was likely to be as a result of a low pH gonad environment, characteristic of many marine invertebrates (Chia and Bickell 1983), which might contribute to OA tolerance for sperm. Furthermore, the release of egg‐derived compounds under OA can promote sperm motility (Ward et al. 1985; Darszon et al. 2008) and perhaps stimulate fertilization. Gonad environment and egg‐derived compounds could explain why the negative effect of OW on hatching event success was lessened by combined exposure to OA conditions in *O. labronica*.

### The implications of trans‐generational experiments for their use in conservation or restoration

Ocean warming and acidification is taking place at an unprecedented pace (IPCC, 2013) and there is concern that natural rates of acclimation and adaptation may be insufficient for organisms to keep up. The human‐assisted, trans‐generational exposure of marine organisms to future ocean conditions has therefore been suggested as one possible tool to improve the tolerance, and thus persistence, of subsequent generations (Van Oppen et al. 2015). Laboratory experiments such as ours can control the strength and type of environmental pressure, enabling significant adjustments to occur trans‐generationally, potentially preparing organisms to respond more effectively to future environmental change.

Within‐generational exposure to OA reduced the negative effects of OW and *vice versa*, with no need for trans‐generational improvements in our model species. On the other hand, results from our single‐stressor exposure support the use of trans‐generational effects to improve offspring resilience, as increases in ocean temperature and decreases in pH are likely to vary both geographically and temporally. For example, pronounced warming is expected in the Mediterranean region (Giorgi and Lionello 2008) where our model species was collected, while pH decreases are expected to be greater and occur faster in Polar regions where carbonate saturation states are already low (Orr et al. 2005; Doney et al. 2012). Furthermore, extreme climatic events associated with climate change, such as heat waves or increased rainfall, may result in temporally acute increases in temperature or decreases in pH (Jones et al. 2007). Persistence under, or recovery from, such changes could be aided by the laboratory preconditioning of populations to OW or OA as evidenced by our results.

The mechanism for trans‐generational improvements, however, could have implications for future generations and may be important to consider when choosing the magnitude of environmental stress exposure. For example, trans‐generational selection, as may have occurred under OW in our study, could result in reduced genetic diversity, rendering populations more susceptible to disease and less plastic in response to further changes (Reed and Frankham 2003), while improvements through TGP, as we most likely observed under OA, could retard adaptation (Price et al. 2003; Sunday et al. 2014).

In conclusion, trans‐generational exposure experiments may not be a universally useful tool for all species at risk to global change (see Welch et al. 2014). In such cases, other human‐assisted approaches may be more appropriate (e.g. Griffith et al. 1989; Van Oppen et al. 2015; Aswani et al. 2015). Nevertheless, based on our single‐stressor results, and the majority of previous studies, it is clear that human‐assisted, trans‐generational exposure to future global change conditions could play a valuable role in enhancing conservation and restoration efforts for marine metazoans. Future studies should aim to find which taxa are likely to benefit from human‐assisted trans‐generational effects, whether trans‐generational improvements persist over successive generations and whether there are any costs of such improvements.

## Data archiving statement

Data for this study are available at PANGAEA^®^.

## Supporting information


**Figure S1.** Experimental setup containing F2 individuals of *Ophryotrocha labronica*.

**Figure S2.** Experimental design used in this experiment.

**Table S1.** Seawater parameters for the four experimental conditions: control (C: 27°C, pH 8.05), ocean warming (OW: 30°C, pH 8.05), ocean acidification (OA: 27°C, pH 7.60) and their combination (OWA: 30°C, pH 7.60).

**Table S2**. Mean values ± SE for different life history and physiological traits measured in *O. labronica* following within‐generational (C–OW; C–OA; C–OWA) and trans‐generational (C–C; OW–OW; OA–OA; OWA–OWA) exposure to control (C), ocean acidification (OA), ocean warming (OW) and combined (OWA) conditions.

**Table S3.** Results of General Linear Models investigating the effect of trans‐generational *vs*. within‐generational exposure to ocean warming (OW) conditions in *O. labronica*.

**Table S4.** Results of General Linear Models investigating the effect of trans‐generational *vs*. within‐generational exposure to ocean acidification (OA) conditions in *O. labronica*.

**Table S5.** Results of General Linear Models investigating the effect of trans‐generational *vs*. within‐generational exposure to ocean warming and acidification combined (OWA) conditions in *O. labronica*.
