# Supplementary figures and images for: Can trans‐generational experiments be used to enhance species resilience to ocean warming and acidification?
Source: Evol Appl. 2016 Jul 6;9(9):1133–46. doi: 10.1111/eva.12391 (PMC5039326; doi:10.1111/eva.12391)

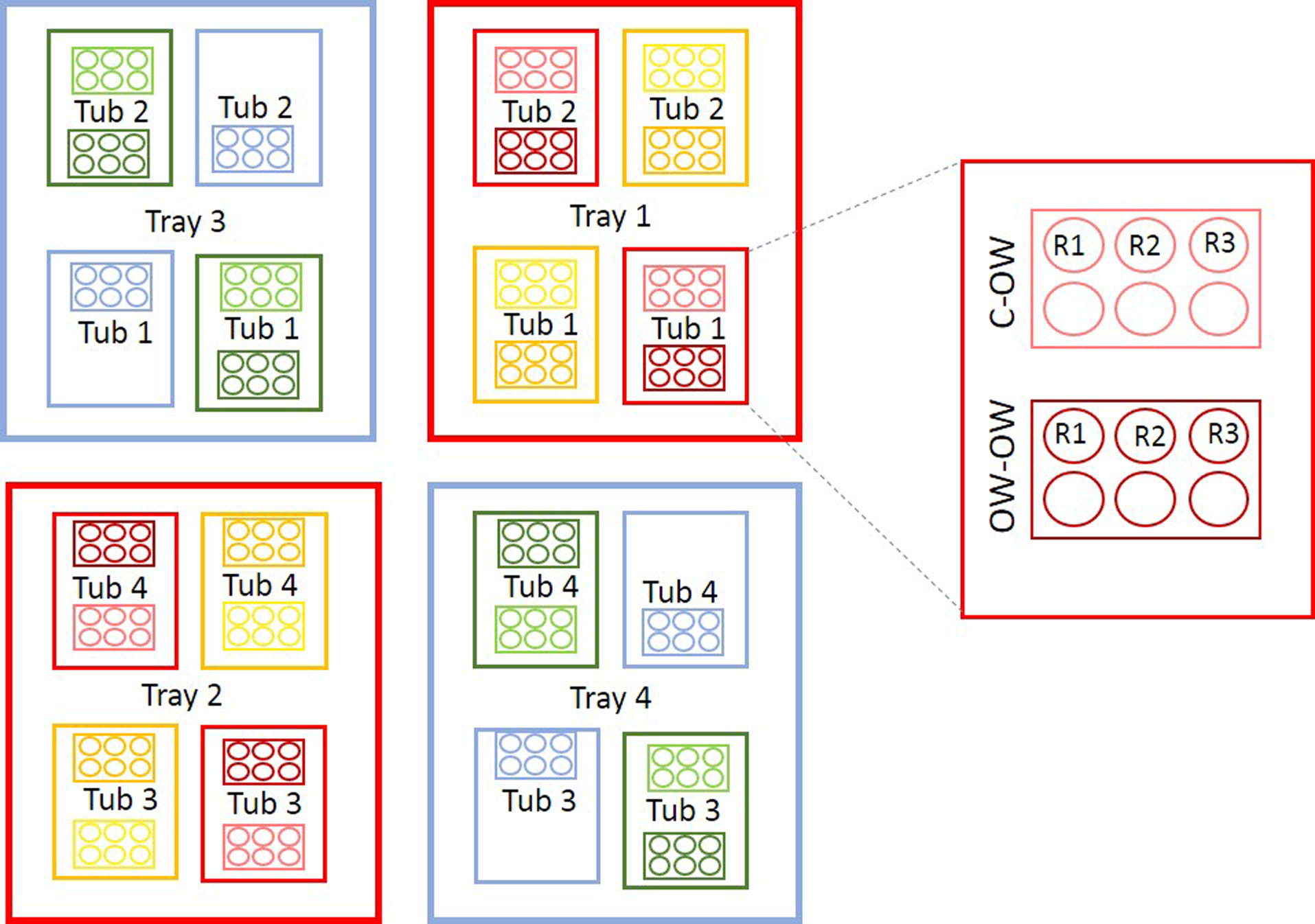

Supplement: Supplementary file 1 — Figure S1. Experimental setup containing F2 individuals of Ophryotrocha labronica. [file EVA-9-1133-s001.tif]

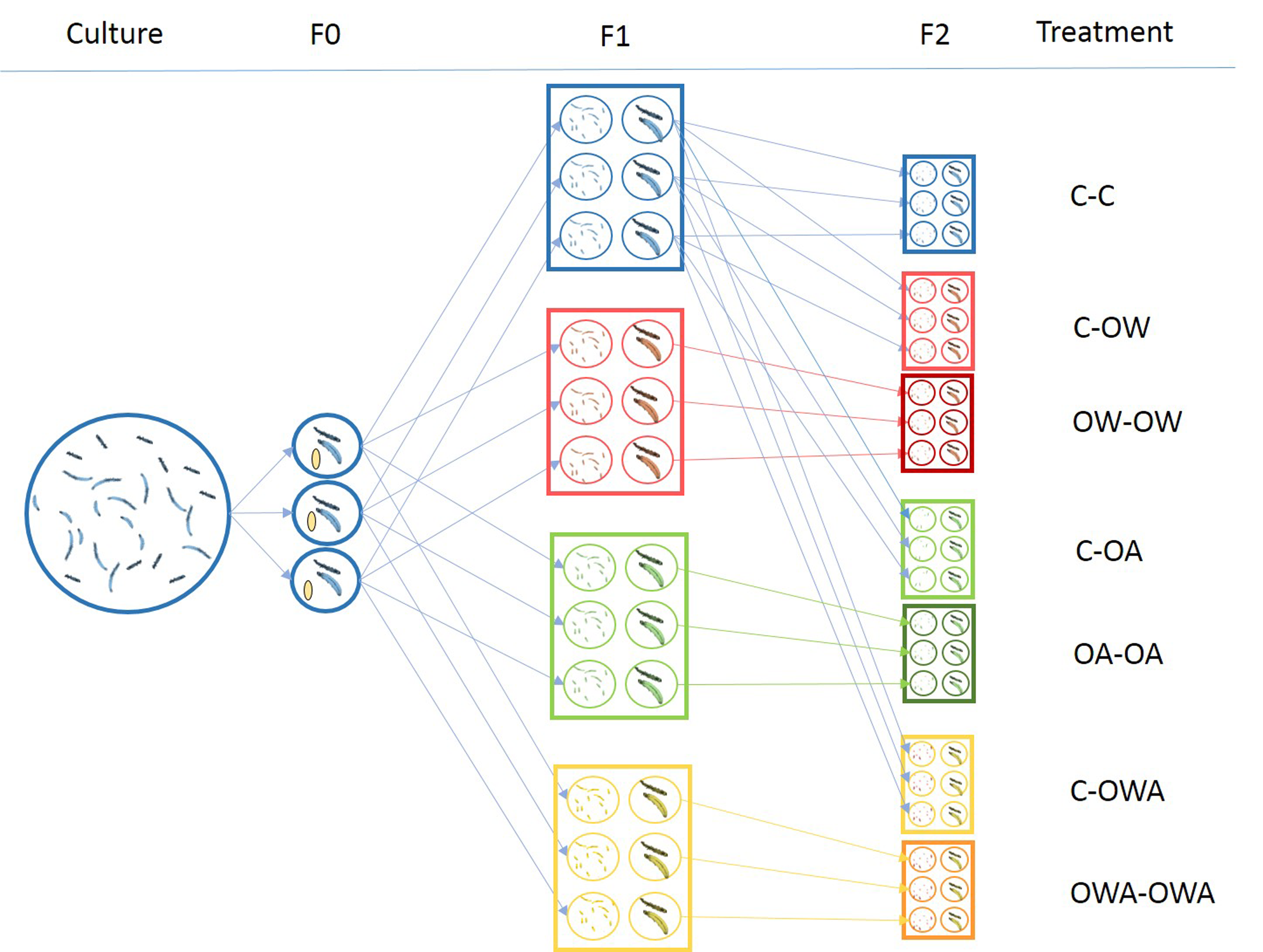

Supplement: Supplementary file 2 — Figure S2. Experimental design used in this experiment. [file EVA-9-1133-s002.tif]

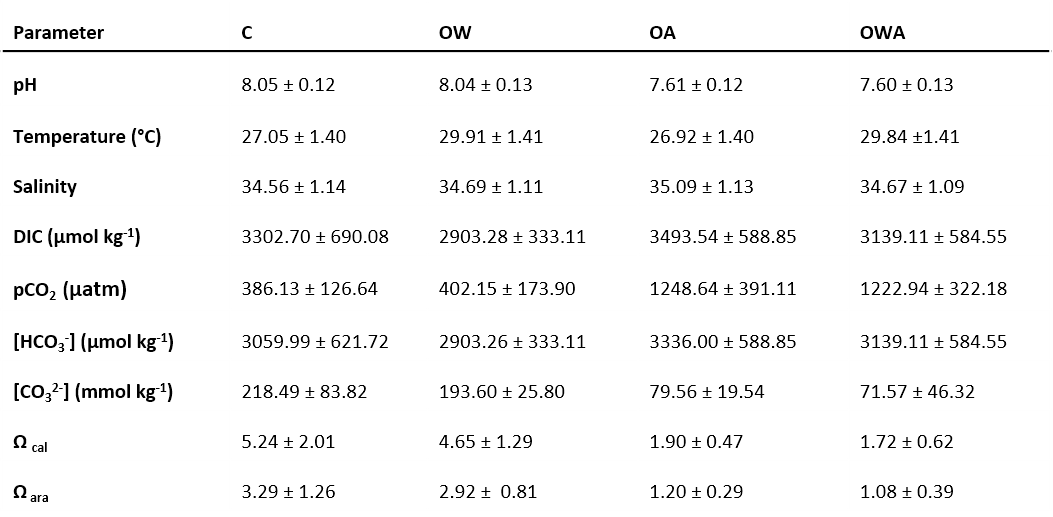

Supplement: Supplementary file 3 — Table S1. Seawater parameters for the four experimental conditions: control (C: 27°C, pH 8.05), ocean warming (OW: 30°C, pH 8.05), ocean acidification (OA: 27°C, pH 7.60) and their combination (OWA: 30°C, pH 7.60). [file EVA-9-1133-s003.tif]

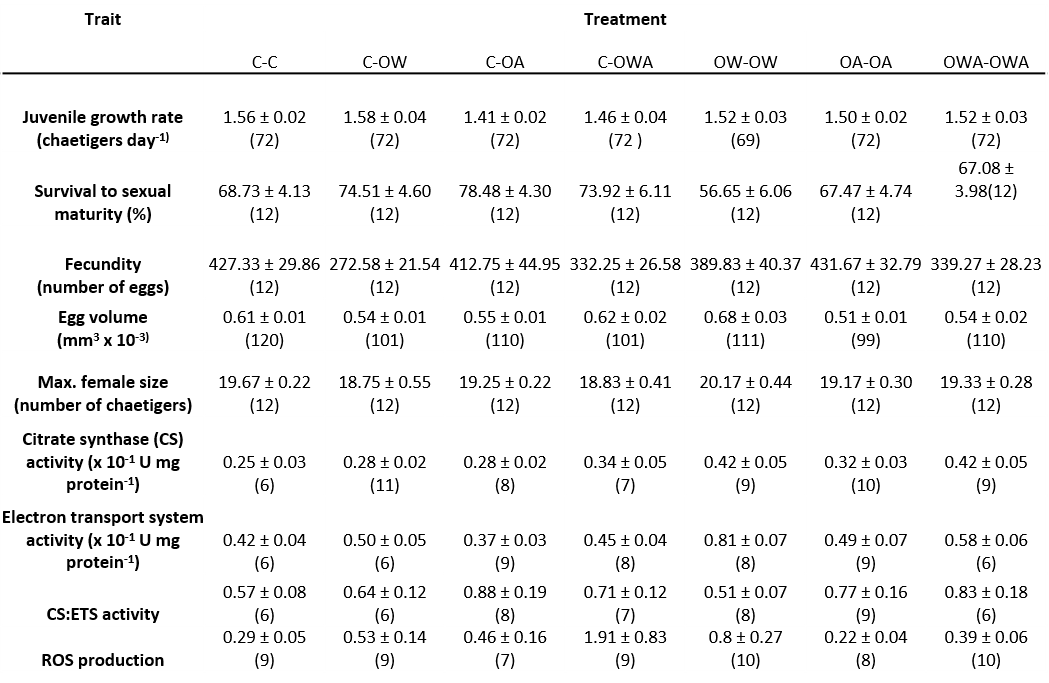

Supplement: Supplementary file 4 — Table S2. Mean values ± SE for different life history and physiological traits measured in O. labronica following within‐generational (C–OW; C–OA; C–OWA) and trans‐generational (C–C; OW–OW; OA–OA; OWA–OWA) exposure to control (C), ocean acidification (OA), ocean warming (OW) and combined (OWA) conditions. [file EVA-9-1133-s004.tif]

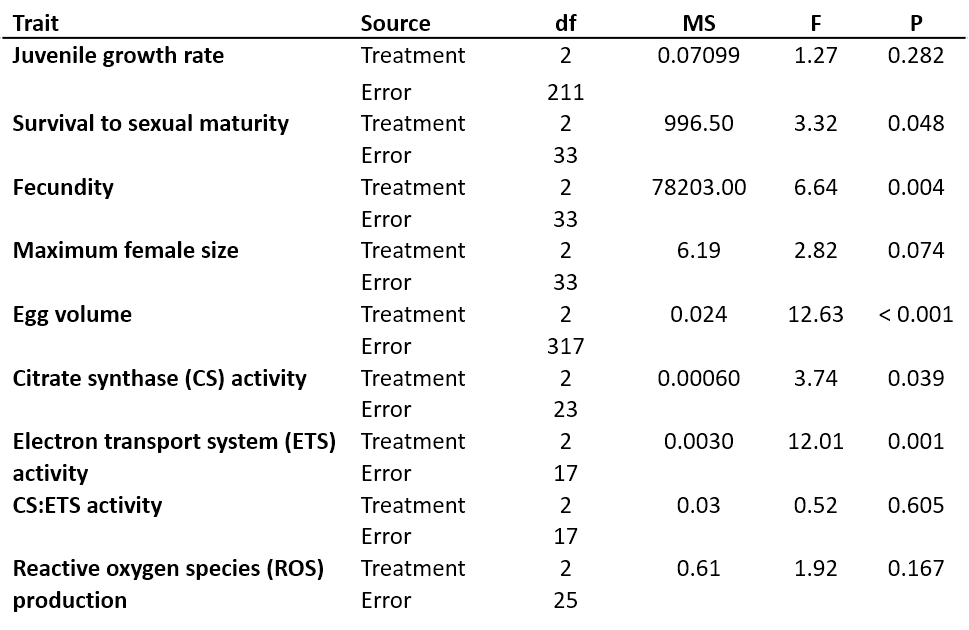

Supplement: Supplementary file 5 — Table S3. Results of General Linear Models investigating the effect of trans‐generational vs. within‐generational exposure to ocean warming (OW) conditions in O. labronica. [file EVA-9-1133-s005.tif]

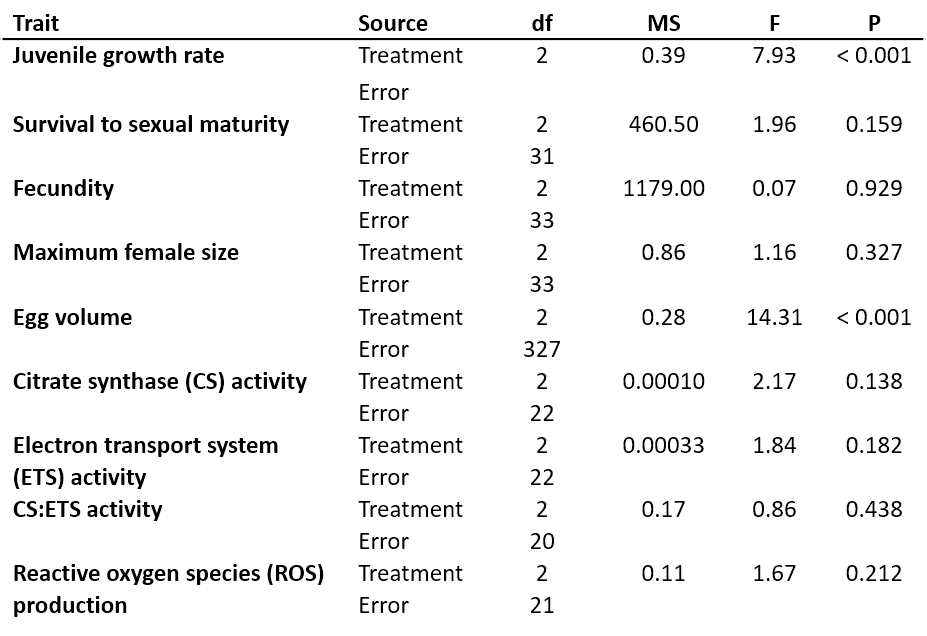

Supplement: Supplementary file 6 — Table S4. Results of General Linear Models investigating the effect of trans‐generational vs. within‐generational exposure to ocean acidification (OA) conditions in O. labronica. [file EVA-9-1133-s006.tif]

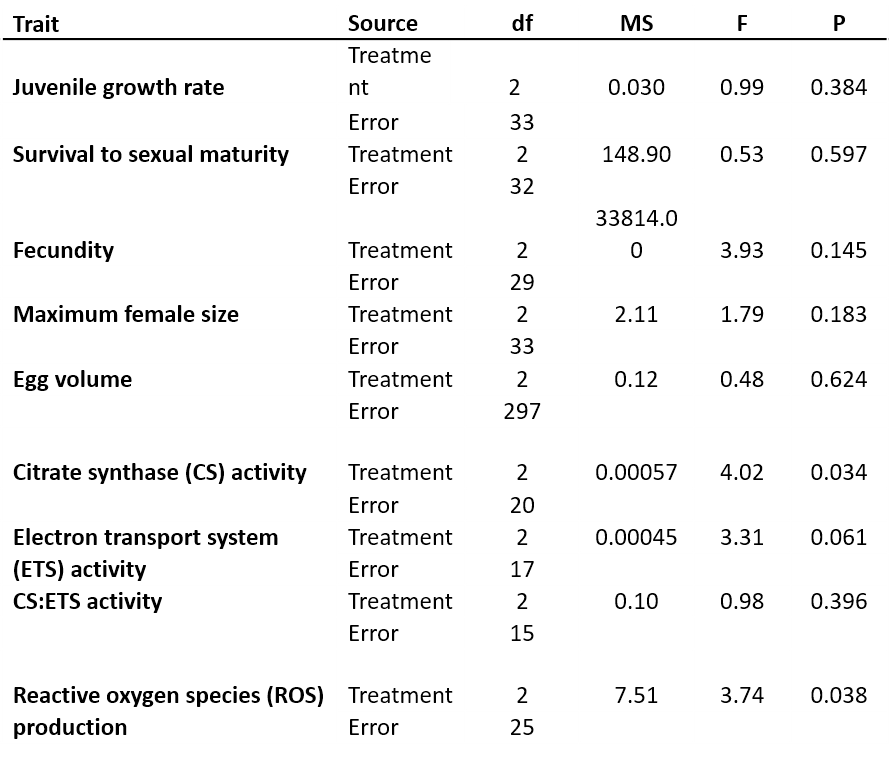

Supplement: Supplementary file 7 — Table S5. Results of General Linear Models investigating the effect of trans‐generational vs. within‐generational exposure to ocean warming and acidification combined (OWA) conditions in O. labronica. [file EVA-9-1133-s007.tif]
