# Supplementary material for: Can trans‐generational experiments be used to enhance species resilience to ocean warming and acidification?
Source: Evol Appl. 2016 Jul 6;9(9):1133–46. doi: 10.1111/eva.12391 (PMC5039326; doi:10.1111/eva.12391)
Supplement: Supplementary file 8 [file EVA-9-1133-s008.docx]

Figure S1. Experimental setup containing F2 individuals of Ophryotrocha

labronica.

Figure S2. Experimental design used in this experiment.

Table S1. Seawater parameters for the four experimental conditions:

control (C: 27°C, pH 8.05), ocean warming (OW: 30°C, pH 8.05), ocean

acidification (OA: 27°C, pH 7.60) and their combination (OWA: 30°C,

pH 7.60).

Table S2. Mean values _ SE for different life history and physiological

traits measured in *O.* labronica following

within-generational (C–OW; C–OA; C–OWA) and transgenerational

(C–C; OW–OW; OA–OA; OWA–OWA) exposure to control

(C), ocean acidification (OA), ocean warming (OW) and combined

(OWA) conditions.

Table S3. Results of General Linear Models investigating the effect of

trans-generational vs. within-generational exposure to ocean warming

(OW) conditions in O. labronica.

Table S4. Results of General Linear Models investigating the effect of

trans-generational vs. within-generational exposure to ocean acidification

(OA) conditions in O. labronica.

Table S5. Results of General Linear Models investigating the effect of

trans-generational vs. within-generational exposure to ocean warming and acidification combined (OWA) conditions in O. labronica.
